# Supplementary material for: A UMLS-based spell checker for natural language processing in vaccine safety
Source: BMC Med Inform Decis Mak. 2007 Feb 12;7:3. doi: 10.1186/1472-6947-7-3 (PMC1805499; doi:10.1186/1472-6947-7-3)
Supplement: Additional file 13 — RAP application directory. Sets up directory for RAP (RDF application for PHP) source code [file 1472-6947-7-3-S13.gz › rap/doc/phpdoc/__filesource/fsource_resModel__resModelResContainer.php.html]

xml version="1.0" encoding="iso-8859-1"?


File Source for ResContainer.php


# Source for file ResContainer.php

Documentation is available at ResContainer.php

```
1. <?PHP
2. // ----------------------------------------------------------------------------------
3. // Class: ResContainer
4. // ----------------------------------------------------------------------------------


8. /**
9. * An RDF Container.
10. * This Class defines methods for accessing RDF container resources.
11. * These methods operate on the RDF statements contained in a model.
12. *
13. * <BR><BR>History:<UL>
14. * <LI>10-01-2004                : First version of this class.</LI>
15. *
16. * @version  V0.9.1
17. * @author Daniel Westphal <mail at d-westphal dot de>
18. *
19. * @package     resModel
20. * @access    public
21. ***/

23. class ResContainer extends ResResource
24. {
25. /**
26. * Holds a ResResource of this container type rdf:seq, rdf:alt, or rdf:bag
27. * @var        ResResource
28. * @access    private
29. */
30. var $containerType;


33. /**
34. * Constructor
35. * You can supply a URI
36. *
37. * @param string $uri
38. * @access    public
39. */
40. function ResContainer($uri = null)
41. {
42. parent::ResResource($uri);
43. }

45. /**
46. * Add a new value to a container.
47. * The new value is added as the last element of the container.
48. *
49. * @param    object ResResource/ResLiteral    $object
50. * @access    public
51. */
52. function add($object)
53. {
54. //type this container, if it isn't already typed
55. if(!$this->hasProperty(new ResResource(RDF_NAMESPACE_URI.RDF_TYPE)))
56. $this->addProperty(new ResResource(RDF_NAMESPACE_URI.RDF_TYPE),$this->containerType);
57. //get the current size
58. $actualSize=$this->size();
59. //add the object to the last position
60. $this->addProperty(new ResResource(RDF_NAMESPACE_URI.'_'.($actualSize+1)),$object);
61. }

63. /**
64. * Determine whether the container contains a value
65. *
66. * @param    obejct ResResource/ResLiteral    $resResource
67. * @return    boolean
68. * @access    public
69. */
70. function contains($resResource)
71. {
72. //get all container's properties
73. foreach ($this->listProperties() as $statement)
74. {
75. //if the property matches a container membership property
76. if ($this->_predicateLabelMatchesMembershipProperty($statement->getLabelPredicate()))
77. {
78. //check, if it's the value, we're looking for.
79. if ($resResource->equals($statement->getObject()))
80. return true;
81. }
82. }
83. return false;
84. }

86. /**
87. * Returns true, if this resource is a container from type rdf:alt
88. *
89. * @return    boolean
90. * @access    public
91. */
92. function isAlt()
93. {
94. return ($this->containerType->getURI()==RDF_NAMESPACE_URI.RDF_ALT);
95. }

97. /**
98. * Returns true, if this resource is a container from type rdf:bag
99. *
100. * @return    boolean
101. * @access    public
102. */
103. function isBag()
104. {
105. return ($this->containerType->getURI()==RDF_NAMESPACE_URI.RDF_BAG);
106. }

108. /**
109. * Returns true, if this resource is a container from type rdf:seq
110. *
111. * @return    boolean
112. * @access    public
113. */
114. function isSeq()
115. {
116. return ($this->containerType->getURI()==RDF_NAMESPACE_URI.RDF_SQE);
117. }

119. /**
120. * Get an array of all resources that are values of this container
121. *
122. * @return    array
123. * @access    public
124. */
125. function getMembers()
126. {
127. $return=array();
128. foreach ($this->listProperties() as $statement)
129. {
130. $predicateLabel=$statement->getLabelPredicate();
131. if ($this->_predicateLabelMatchesMembershipProperty($predicateLabel))
132. {
133. $return[$this->_getMemberIndexNrFromMembershipPropertyLabel($predicateLabel)] = $statement->getObject();
134. }
135. }
136. return $return;
137. }

139. /**
140. * Remove a value from the container.
141. *
142. * Once removed, the values in the container with a higher ordinal value are renumbered.
143. * The renumbering algorithm depends on the type of container.
144. *
145. * @param    obejct ResResource/ResLiteral    $resResource
146. * @access    public
147. */
148. function remove($object)
149. {
150. $deleteFromIndex=array();
151. //get all container members
152. $memberIndex=$this->getMembers();

154. //check each container member if it equals the resoure to be removed
155. foreach ($memberIndex as $key => $value)
156. {
157. //save the statements positio in the container
158. if($object->equals($value))
159. $deleteFromIndex[]=$key;
160. }

162. //delete all found container members
163. foreach ($deleteFromIndex as $index)
164. {
165. $this->removeAll($this->_getMembershipPropertyWithIndex($index));

167. //renumber all members with higher ordinal numbers than the deleted one
168. for ($i = $index;$i < count($memberIndex); $i++)
169. {
170. $this->removeAll($this->_getMembershipPropertyWithIndex($i+1));
171. $this->addProperty($this->_getMembershipPropertyWithIndex($i),$memberIndex[$i+1]);
172. }
173. }

175. }

177. /**
178. * Returns the number values in the container.
179. *
180. * @return    integer
181. * @access    public
182. */
183. function size()
184. {
185. return count($this->getMembers());
186. }

188. /**
189. * Checks, if a predicate label fits a container membership property rdf:_n
190. *
191. * @param    string    $predicateLabel
192. * @return    boolean
193. * @access    private
194. */
195. function _predicateLabelMatchesMembershipProperty($predicateLabel)
196. {
197. return substr($predicateLabel,0,strlen(RDF_NAMESPACE_URI.'_')) == RDF_NAMESPACE_URI.'_';
198. }

200. /**
201. * Get the ordinal number from a membership property rdf:_n
202. *
203. * @param    string    $predicateLabel
204. * @return    integer
205. * @access    private
206. */
207. function _getMemberIndexNrFromMembershipPropertyLabel($predicateLabel)
208. {
209. return (int)substr($predicateLabel,strlen(RDF_NAMESPACE_URI.'_'));
210. }

212. /**
213. * Get a membership property rdf:_n with index $int
214. *
215. * @param    intger    $int
216. * @return    string
217. * @access    private
218. */
219. function _getMembershipPropertyWithIndex($int)
220. {
221. return new ResResource(RDF_NAMESPACE_URI.'_'.$int);
222. }
223. }
224. ?>
```

Documentation generated on Thu, 7 Jul 2005 13:43:31 +0200 by phpDocumentor 1.3.0RC3
